# Radiographic assessment of maxillary sinus membrane and lateral wall thickness using cone-beam CT in different facial types in southwestern Saudi Arabia

**DOI:** 10.1371/journal.pone.0298403

**Published:** 2024-03-25

**Authors:** Shahabe Saquib Abullais, Saad M. AlQahtani, Salman Alqahtani, Abdulrahman Alaamri, Ali Azhar Dawasaz, Abdulelah Alqahtani, Prasad V. Dhadse

**Affiliations:** 1 Department of Periodontics and Community Dental Sciences, King Khalid University, Abha, Saudi Arabia; 2 Restorative Resident, College of Dentistry, King Khalid University, Abha, Saudi Arabia; 3 Department of Diagnostic Dental Sciences, King Khalid University, Abha, Saudi Arabia; 4 College of Dentistry, King Khalid University, Abha, Saudi Arabia; 5 Department of Periodontology, Datta Meghe Institute of Higher Education and Research Sawangi, Wardha, India; University of Puthisastra, CAMBODIA

## Abstract

The anatomy of the edentulous posterior maxilla and maxillary sinus possess unique challenges in implant dentistry. The purpose of this study was to assess maxillary sinus membrane thickness (MT) and lateral wall thickness (LWT) in different facial index profiles and to describe the clinical implications. A retrospective image analysis of 75 CBCT scans was done, which yielded a total of 150 sinus images. The facial index was calculated as per the formula given in the text and grouped as euryprosopic, mesoprosopic and leptoprosopic. The images obtained were of 36 women (48%) and 39 men (52%), with maximum subjects in 30–39 years age group. MT and LWT were measured at three different points on the radiograph at every 3mm from the base of the sinus floor in premolar and molar regions of each image. Results showed females had significant differences from males in LWT in both premolar and molar regions (p = 0.018 and 0.032 respectively). Subjects in 40–49 years of age had significant differences (p = 0.021) in MT in premolar region only. Also, difference in MT in premolar and molar regions were also statistically significant. Lastly, the present study did not find any statistically significant difference in MT and LWT in all three facial indices groups. It can be concluded that different facial indices have no positive correlation with maxillary sinus membrane thickness and lateral wall thickness. Hence, surgical complications are avoidable with proper detailed knowledge and appropriate identification of the anatomic structures characteristic to the maxillary sinus.

## Introduction

An adequate amount of bone is needed surrounding implants for successful long standing clinical outcomes [1]. Inadequate alveolar ridge height and maxillary sinus dimensions are the key limiting variables that make reconstruction of the posterior maxilla more difficult [2]. Osseo-integrated implants are being used more frequently to restore a functional dentition, but posterior implant placement might be challenging due to insufficient bone height. The preferred treatment for this condition is maxillary sinus (MS) floor elevation surgery [3].

Maxillary Sinus floor augmentation involving Schneiderian membrane elevation through lateral and transcrestal approach has increasingly become an imperative procedure for filling residual crestal bone height in the posterior maxilla in order to increase bone volume for dental implant insertion [4]. Elevation of the maxillary sinus floor is considered a successful and conventional approach for implant placement by augmenting maxillary posterior height, providing dentists with increased bone volume [5–7]. The approach through the lateral wall and crestal regions are the methods for maxillary sinus augmentation that are most frequently employed. Boyne and James were the first to describe the lateral wall technique, followed by Tatum in 1986 [8, 9]. When there is minimum alveolar ridge height in a patient, this approach yields more predictable results [10]. Despite the fact that this procedure has been shown to be highly efficient and predictable, a wide range of complications have been reported during the surgical procedure or in the post-surgical period [11]. As a result, clinicians should be aware of the difficulties and how to manage them.

During the sinus elevation surgery, perforation of the Schneiderian membrane is considered to be the most common intraoperative complication occurring in 11% to 58.3% or an average of up to 19.5% of surgeries [5]. Nevertheless, membrane perforation is reported due to inadequate surgical planning or maneuvers [11, 12].

According to reports, the likelihood of membrane perforations is closely proportional to the thickness of the sinus membrane (MT) [3]. In addition, lateral wall thickness (LWT) of the sinus is also crucial in the lateral window technique for sinus augmentation. Therefore, precision pre-surgical radiographic assessment utilizing cone-beam computed tomography (CBCT) is critical to avoiding Schneiderian membrane perforation.

The most common applications for cone-beam computed tomography (CBCT) include evaluation of dental implant site, traumatic injuries of the oral and maxillofacial region and orthodontic assessment of maxillomandibular jaw relationships [13]. CBCT is recommended as radiographic imaging in a multiplanar forma before treatment planning for MS elevation. It provides crucial diagnostic information at a reduced radiation risk than multislice computed tomography [14].

In maxillary sinus imaging, the normal mucosa on the inner surface of the sinus walls appears as a thin and smooth marginal soft tissue density. Most authors consider lining thickening more than 2-3mm to be mucosal thickening. However, only 40% of people had a clean maxillary sinus with normal thickness mucosa. The lateral wall is thin and continuous inferiorly with the buccal portion of the alveolar ridge when oriented posterolaterally towards the infratemporal fossa. The posterior superior alveolar canal is located in the lateral wall and is an important component to consider when determining the location and design of a flap for maxillary sinus lift procedures [15].

The facial index is a vital classification used by plastic surgeons for the reconstruction of the face [16]. The first reported classification was by Martin in 1928 [17] who described facial indices as euryprosopic, mesoprosopic and leptoprosopic.

The present study was aimed to correlate maxillary sinus membrane thickness (MT) and lateral wall thickness (LWT) with facial index profile with a null hypothesis stating that there is no difference in maxillary sinus membrane and lateral wall thicknesses in different facial index profiles.

## Material and methods

**Fig 1:**
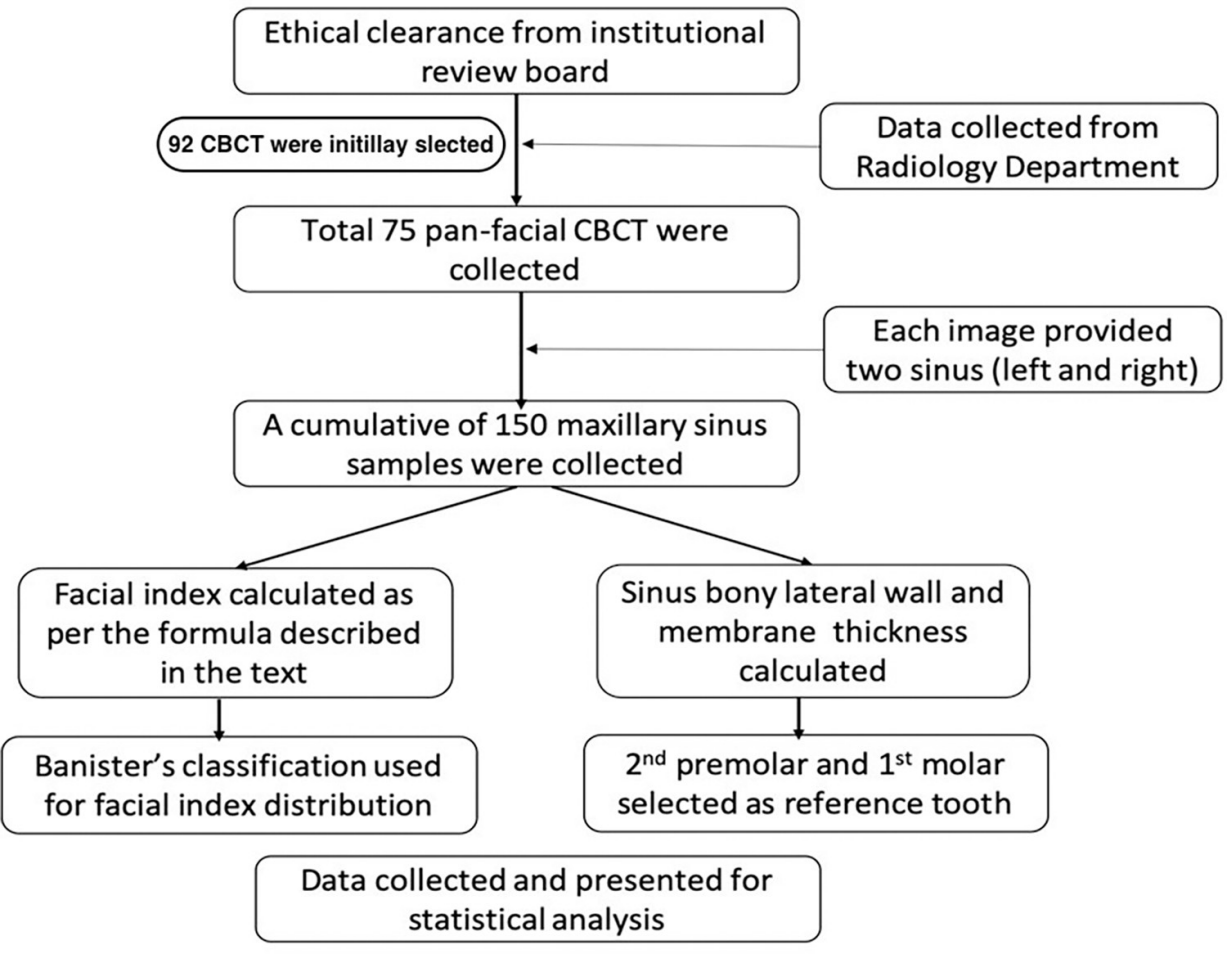
Study flow chart.

The present retrospective radiographic image analysis study was approved by the institutional ethical review board from the College of Dentistry (IRB/KKUCOD/ETH/2019-20/074), King Khalid University, Abha, KSA. The CBCT images used in the current study were recorded from the Kavo 3D Pro, USA imaging system at the Radiology Division, Faculty of Dentistry, King Khalid University. Each image was acquired using the following parameters: 89 kVp; 9.0 mA and 16 sec exposure. The CBCTs taken between 12^th^ November 2019 to 20^th^ December 2020 were included in the study. The data for research purposes were accessed on 2^nd^ September 2020 and collection was completed on 30 December 2020. Sample size was calculated using PS: Power and Sample Size Calculation software ver. 3.1.2. The level of significance (α) was set at 0.05 and power (β) was set at 80%. A total of 150 maxillary sinuses from 75 subjects (40 males and 35 females with a mean age of 32.62±14.22 years, ranging from 20–60 years) were evaluated. In all the patients, CBCT was medically justified, and written informed consent was obtained from all the patients before exposure to the radiation. All patients were informed that their medical and radiological records would be used for research by keeping their identities anonymous. The authors had access to identifying information during or after data collection, but the data were deidentified while recording the study parameters. The study plan is given in the form of a flow diagram as shown in Fig 1.

The patients were included with the following criteria; 1) Saudi nationals, 2) Intact maxillary sinus between first premolar and second molar. 3) Presence of healthy maxillary second premolar and first molar. The patients were excluded from the study if; 1) Unclear or incomplete scan images because of radaiton scattering or other causes. 2) Presence of sinus pathology. 3) Patients with a history of heavy cigarette smoking (more than 20 cigarettes per day) [18], 4) Sinus received bone augmentation or presence of dental implant. 5) Any history of trauma related to sinus.

**Fig 2:**
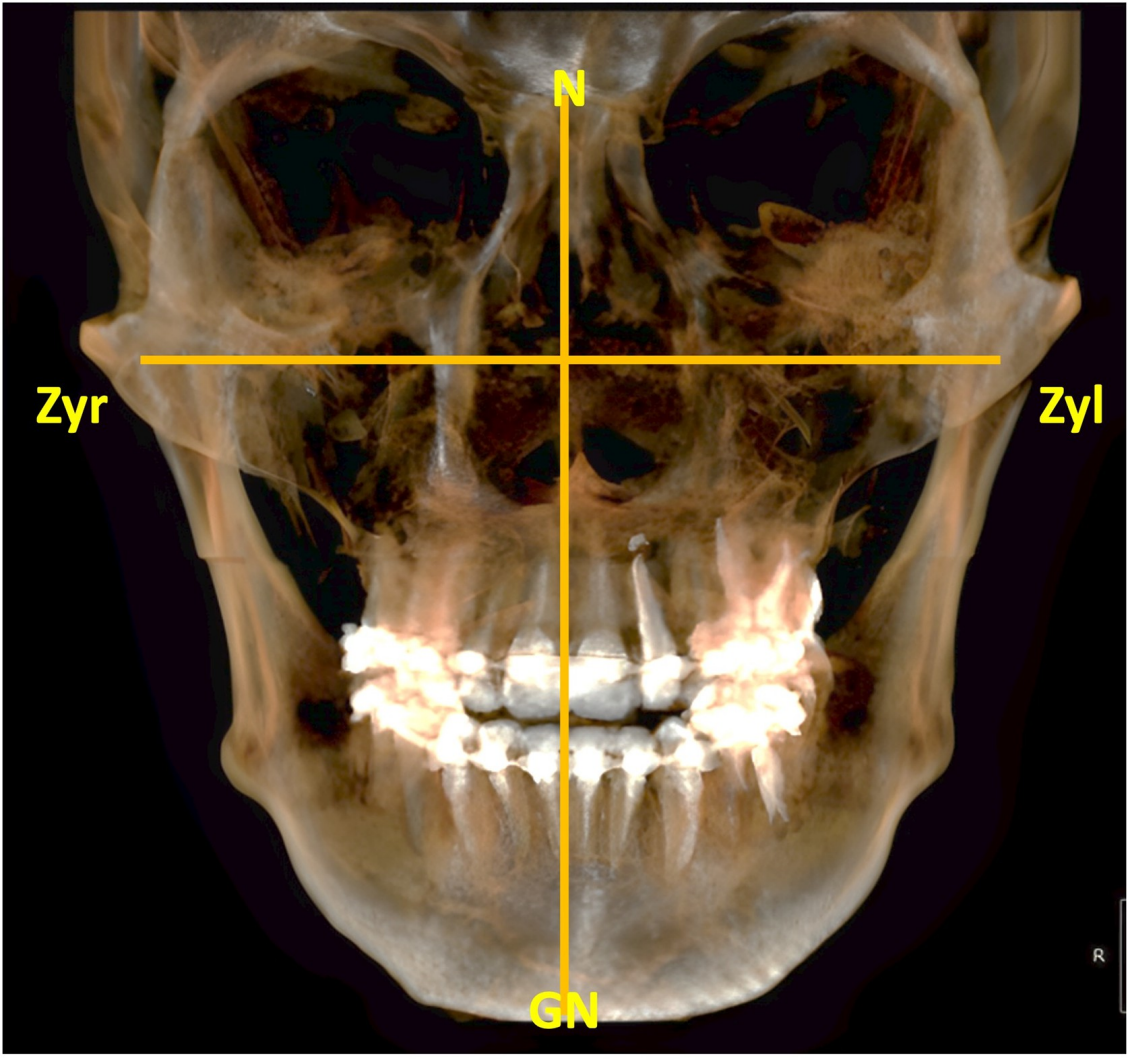
Panfacial CBCT image to calculate facial index.

Three examiners (SA, Ab Al, Ab Al) did the measurements twice at a time gap of 10 days to govern inter-observer variability. All the recorded images were examined using Cliniview^TM^ (Version 11.10.1, 3DOnDemand, GE Healthcare®, Finland) by means of the multiplanar reconstruction module under standardized conditions at an examination terminal. The facial index was determined by using the following formula [19] (Fig 2).


$$Facial\;index=\frac{Morphological\;facial\;height\;(N-Gn)}{Bizygomatic\;width\;(Zyr-Zyl)}\times 100$$

The parameters in the above calculation were defined as follows: 1) Nasion (N): midpoint of the nasofrontal suture. 2) Gnathion (Gn): lowest point on the lower border of the mandible in the midline. 3) Zygion (Zyl and Zyr): it is the most lateral point on the zygomatic arch (left and right).

**Fig 3:**
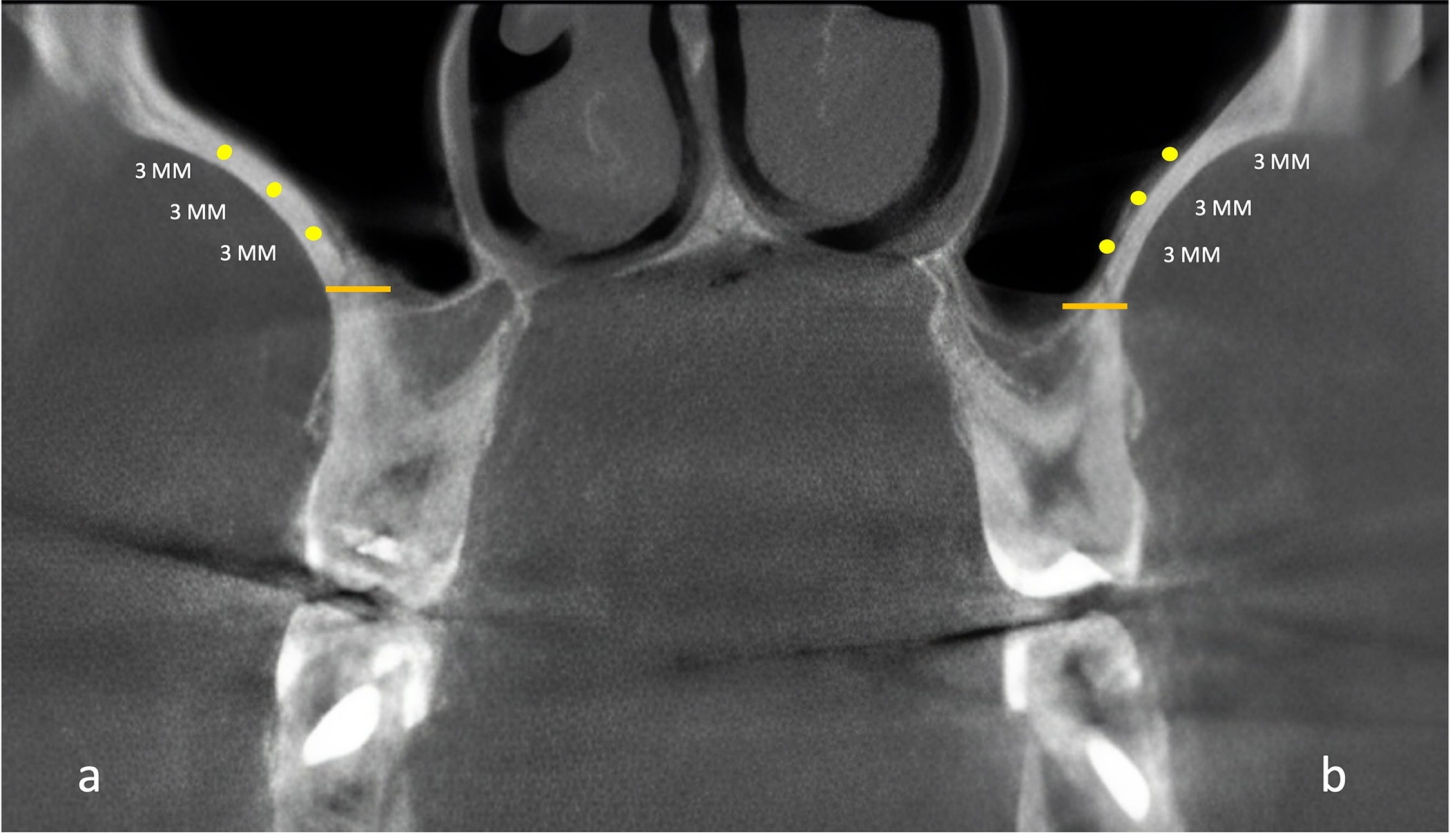
Coronal section of CT scan at the level of maxillary sinus. (A) reference point on lateral wall at 3mm distance form base of the sinus floor, (B) reference point on sinus membrane at 3mm distance from base of the sinus floor).

The calculated value was categorized according to the classification proposed by Bannister in 1928 and published by Williams et. al. [20] as: i) hypereuryprosopic (<79%) ii) euryprosopic (80.0%-84.9%), iii) mesoprosopic (85.0%-89-9%), iv) leptoprosopic (90.0%-94.9%) and v) hyperleptoprosopic (>95%). Maxillary lateral wall and sinus membrane thickness were measured at 2^nd^ premolar and 1^st^ molar teeth. To maintain the standardization, three points at a regular distance of 3 mm on the lateral wall and sinus membrane were made from the base of the maxillary sinus (Fig 3).

The collected data were organized and entered in Microsoft Excel software (version 2010, Microsoft^®^, Redmond, USA), and analyzed by using Statistical Package for the Social Sciences (SPSS, v.20, IBM, Chicago, USA). Mean and standard deviation values were calculated. Chi-square test was used to compare two categorical data in a contingency table. Frequency tables were used to determine the proportion level of variables among surveyed patients, with the significance level set at p<0.05.

## Results

**Fig 4:**
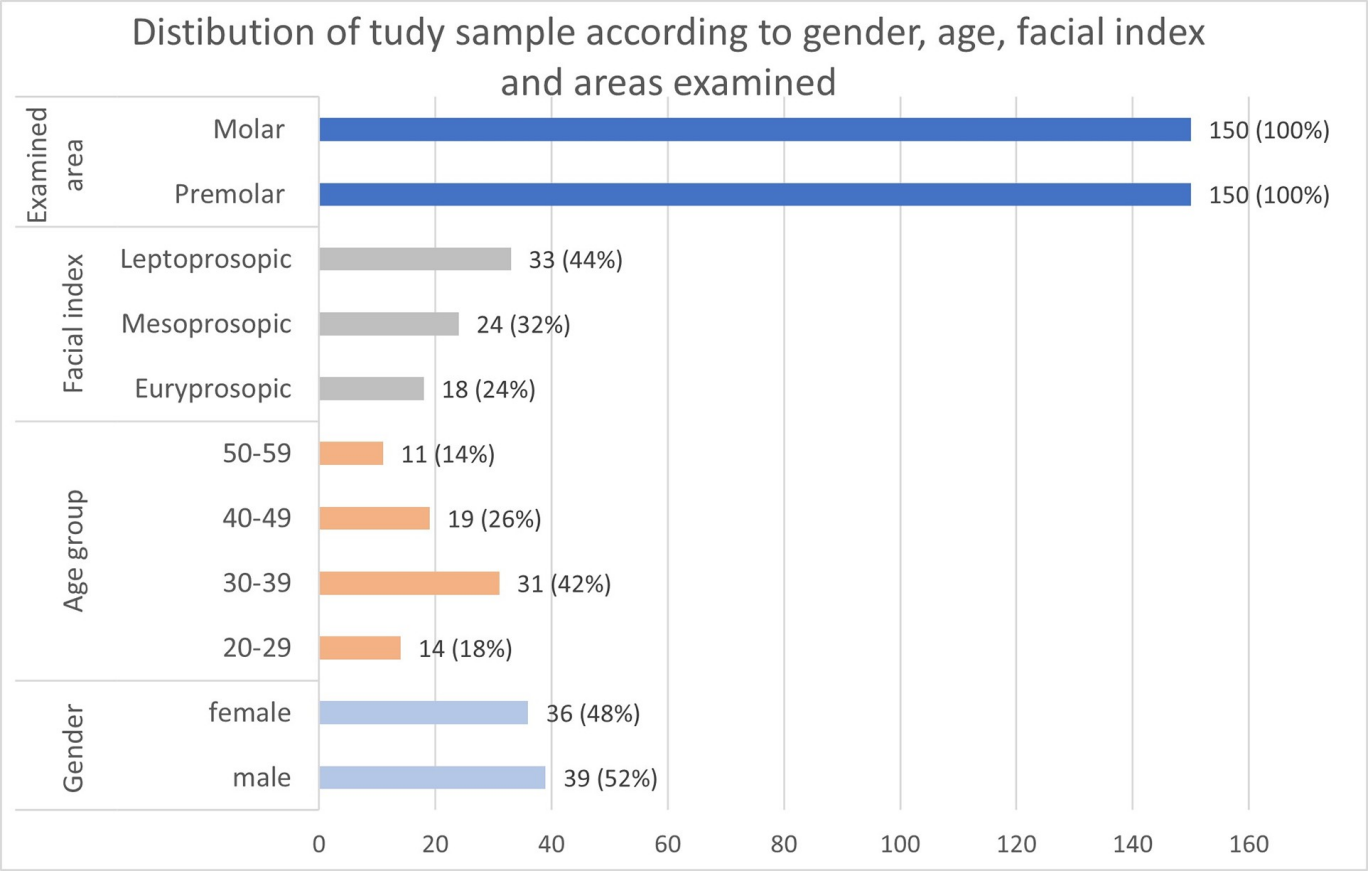
Distibution of study sample according to areas examined, facial index, age in years, and gender.

A total of 92 CBCT were examined for the sample selection, however 17 out of 92 scans were excluded since they exhibited pathologic changes or unsharp appearance. Finally, 75 good quality images were included in the study, which yielded 150 maxillary sinus images. The sample included an almost equal distribution of males and females with a mean age of 32.62±14.22 years. Depending on the type of facial index, almost half of the scans belong to leptoprosopic type followed by mesoprosopic and euryprosopic. None of the subjects had hypereuryprosopic and hyperleptoprosopic facial types (Fig 4).

The distribution of mean maxillary sinus membrane thickness in the region of premolar and molar did not differ significantly in male and female subjects (P-value>0.05). Whereas, mean maxillary sinus lateral wall thickness in the region of premolar and molar is significantly higher in females compared to males (P-value<0.05) (Table 1).

**Table 1:** Distribution of mean sinus membrane and lateral wall thickness according to sex of the participants.

		Membrane thickness		Lateral wall thickness	
Sex	n	Pre molar	Molar	Pre molar	Molar
		Mean± SD	Mean± SD	Mean± SD	Mean± SD
Male	39	1.49±0.51	1.23±0.49	1.54±0.50	1.56±0.59
Female	36	1.43±0.47	1.11±0.57	1.83±0.56	1.890±0.74
p-value		0.617^NS^	0.327^NS^	0.018*	0.032*

p-value by independent sample t test. p-value<0.05 is considered to be statistically significant.

NS-Statistically non-significant.

Age-wise analysis showed significant differences in the mean sinus membrane thickness (P-value = 0.021) in premolar region as shown in Table 2. However, MT in molar region and LWT in premolar as well as molar regions did not show statistically significant differences in various age groups. Additionally, 40–49 year-old subjects had the least thickness of MT and LWT.

**Table 2:** Distribution of mean sinus membrane and lateral wall thickness according to age group of cases studied.

		Membrane thickness		Lateral wall thickness	
Age group	*n*	Pre molar	Molar	Pre molar	Molar
		Mean± SD	Mean± SD	Mean± SD	Mean± SD
20–29	14	1.57±0.43	1.19±0.47	1.87±0.62	1.74±0.68
30–39	31	1.50±0.52	1.24±0.43	1.6662	1.56±0.64
40–49	19	1.17±0.40	1.15±0.64	1.58±0.39	.86±0.77
50–60	11	1.77±0.47	1.05±0.52	1.61±0.47	1.91±0.62
P-value		0.021*	0.754^NS^	0.464^NS^	0.478^NS^

P-value by ANOVA. P-value<0.05 is considered to be statistically significant.

*P-value<0.05

NS-Statistically non-significant.

On categorizing the results in terms of facial index, it is observed in Table 3 that the mean maxillary membrane thickness and sinus lateral wall thickness in the region of molar and premolar did not differ significantly across various groups of cases studied (P-value>0.05).

**Table 3:** Distribution of mean sinus membrane and lateral wall thickness according to facial index of cases studied.

		Membrane thickness		Lateral wall thickness	
Facial Index	*n*	Pre molar Mean± SD	Molar	Pre molar	Molar
			Mean± SD	Mean± SD	Mean± SD
Euryprosopic	14	1.42±0.49	1.11±0.47	1.77±0.67	1.66±0.63
Mesoprosopic	31	1.36±0.53	1.02±0.54	1.68.52	1.72±0.73
Leptoprosopic	19	1.56±0.45	1.31±0.54	1.63±0.51	.75±0.69
p-value		0.754^NS^	0.09816^NS^	0.672^NS^	0.899^NS^

p-value by ANOVA. p-value<0.05 is considered to be statistically significant. NS-Statistically non-significant.

Finally, Table 4 shows that mean maxillary sinus membrane thickness differs significantly in premolar and molar regions in the study group (P-value<0.001). Whereas, mean lateral wall thickness did not differ significantly between premolar and molar regions in the study group (P-value>0.05).

**Table 4:** Distribution of mean membrane thickness and mean lateral wall thickness between premolar and molar regions.

	Pre molar	Molar	P-value
**MT and LWT**	Mean± SD	Mean± SD	
**Maxillary sinus membrane thickness**	1.46±0.49	1.17±0.53	0.001**
**Maxillary sinus lateral wall thickness**	1.68±0.55	1.72±0.68	0.676^NS^

p-value by ANOVA. p-value<0.05 is considered to be statistically significant.

**P-value<0.001

NS-Statistically non-significant.

Table 5 shows correlation of MT and LWT in premolar and molar regions in different facial indices. Results showed a significant positive correlation of MT in premolar and molar regions, however, there was a strong negative correlation of MT in molar region and LWT in premolar region. Whereas Table 6 shows MT and LWT in premolar and molar areas correlation with side, sex and age of sample subjects. There was highly significant correlation of LWT in molar areas with right or left side of face (p = 0.009) whereas sex of an individual significantly determined the thickness MT in premolar area (p = 0.016).

**Table 5:** Pearson correlation of membrane thickness and lateral wall thickness according to facial indices.

Correlations						
		Facial index	MT	LWT	MT	LWT
			(Premolar)	(Premolar)	(Molar)	(Molar)
Facial index	Pearson Correlation	1				
	Sig. (2-tailed)					
	N	60				
MT (Premolar)	Pearson Correlation	.056	1			
	Sig. (2-tailed)	.673				
	N	60	120			
LWT (Premolar)	Pearson Correlation	-.126	-.109	1		
	Sig. (2-tailed)	.336	.234			
	N	60	120	120		
MT (Molar)	Pearson Correlation	.054	.534**	-.192*	1	
	Sig. (2-tailed)	.683	.000	.035		
	N	60	120	120	120	
LWT (Molar)	Pearson Correlation	.130	.022	-.033	.075	1
	Sig. (2-tailed)	.321	.815	.719	.412	
	N	60	120	120	120	120

**. Correlation is significant at the 0.01 level (2-tailed).

*. Correlation is significant at the 0.05 level (2-tailed).

**Table 6:** Pearson correlation of membrane thickness and lateral wall thickness according to side, sex and age of sample subjects.

		Membrane thickness Premolar	Lateral Wall thickness Molar	Membrane thickness Molar	Lateral Wall thickness Premolar
Side (Right and Left)	Pearson Correlation	.142	-.238**	.051	-.075
	Sig. (2-tailed)	.121	.009	.578	.414
	N	120	120	120	120
Sex	Pearson Correlation	.219*	-.113	.103	1
	Sig. (2-tailed)	.016	.218	.264	
	N	120	120	120	120
Age	Pearson Correlation	-.128	-.146	.078	1
	Sig. (2-tailed)	.329	.265	.556	
	N	60	60	60	60

**. Correlation is significant at the 0.01 level (2-tailed).

*. Correlation is significant at the 0.05 level (2-tailed).

## Discussion

The maxillary sinus is the largest of the four maxillofacial sinuses that forms a significant portion of the facial region [21] and has thus been the core of research in a variety of clinical fields [13, 15, 16, 21–24]. So far, the majority of investigations on sex and maxillary sinus growth have been clinical case studies [25–27]. Jun BC et al., discovered that the maxillary sinus grows till puberty in girls and into the twenties in males [28]. Given the scarcity of research on this demographic, adult studies are crucial. While most treatments are given to adults, no research have been done to compare the maxillary sinus dimensions to adult facial profile.

Gender, race, social background, nutrition, and genetics all have an impact on the development and structure of the facial skeleton [29]. Angle classification has been employed for facial skeletal measurements, however it evaluates the relationship between the maxillary and mandibular molars, which might not effectively describe the facial profile [29]. In anthropometry, the facial index is used to describe facial proportions. The facial index, which is the percentile ratio of facial height to facial breadth, is used to classify facial forms [19]. Anatomically, there are five types of facial index: hypereuryprosopic face (very broad face), eryprosopic face (broad face) Mesoprosopic (round) face, Leptoprosopic (long face), and Hyperleptoprosopic (long face) face (very long face) [20]. Another study examined the maxillary sinus size and compared it to three facial indices, namely mesoprosopic, leptoprosopic, and hyperleptoprosopic. They concluded that maximum subjects (~70%) had leptoprosopic facial characteristic, similar to the present study that showed almost half of the subjects with similar facial features (44%) [27].

MT and LWT can also influence the placement of dental implants. Prognostic factors for implant therapy include vital factors such as patients’ awareness along with maxillary bone density, and age of the patient [30, 31]. Additionally, increased thickness of the maxillary sinus wall is seen as a potential risk factor in open sinus lift procedures. Because greater thickness complicates and prolongs surgery, knowing the thickness of the maxillary sinus wall allows the surgeon to select sites with a lower thickness to minimize surgical problems such as membrane perforations [11, 23]. A study by Kalyvas D (2018) [32] found mean MT of 1.6±1.2mm and concluded significant gender (p = 0.01) and non-significant age (p = 0.87) differences. They also found significant difference when compared to mesial and distal thickness values. These findings are similar to our study.

In the present study, females showed significantly thicker LWT than males. However, there was no difference in MT according to the sex of an individual. It is generally found that there is a significant difference in anthropometric measurement between males and females [19]. Conclusively, sex differences in lateral wall thickness in the premolar and molar regions were found to be significant, with p-values of 0.018 and 0.032, respectively. However, considering other parameters assessed in the present study, there were no differences observed.

The authors discovered age-related significant differences in sinus membrane thickness in the premolar region, but not in lateral wall thickness. As a result, until the second to sixth decade of life, bony changes in the maxillary sinus are insignificant. Furthermore, patients of various ages may exhibit altered membrane thickness due to commonly occurring sinus pathologies. The thickness of the maxillary sinus lateral wall is also crucial in several surgical procedures, including Caldwell-Luc surgery, Lefort I osteotomy, open sinus lift, facial and jaw bone fracture repair, and mini-screw insertion in orthodontics, as well as the diagnosis of chronic sinusitis [33–35]. A study by Kawakami (2008) concluded that more the cranial the approach for antrostomy, the better augmentation height is achieved during sinus floor elevation [22]. Another systematic review found 98% implant survival after 3 years when the sinus floor elevation window was through a lateral access [5]. Hence LWT is a major determining prognostic factor for such surgeries. A study on thirty dry skulls revealed no significant differences in the thickness of bony anterior wall of maxillary sinus on right and left side [36]. Our results showed thickest wall above the first molar and the least thick in the premolar area. This difference could be attributed to the presence of the zygomatic buttress.

Although the sinus membrane and lateral wall thickness have been studied previously, their relationship to facial indices has yet to be investigated. Lee et al., (2022) [27] investigated and found concluding evidence of the influence of facial indices on maxillary sinus dimensions. However, the present study found no correlation between facial indices and membrane thickness (p>0.05).

According to the investigators’ knowledge, there is no study in the literature that correlates an individual’s facial index with the thickness of the sinus membrane as well as the lateral wall of the maxillary sinus. In the present study, we found no significant difference in membrane thickness between age and gender groups, which was consistent with previous research [37]. However, the female lateral wall was significantly thicker. Furthermore, the premolar area had a significantly thicker sinus membrane than the molar area. Finally, there was no significant correlation between the various facial indices and membrane thickness or lateral wall thickness.

Limitations of the current study include the risk of subjects having higher thicknesses of the sinus membrane wall caused due to asymptomatic chronic sinusitis. Kim et al., 2008 [34] correlated MT with ethmoid sinus and middle turbinate wall thicknesses. Confounding factors include patients selected from high altitudes could possibly have variations in the thickness of MT/LWT.

## Conclusion

Given the limitations of the current study, it is possible to conclude that different facial indices have no positive correlation with maxillary sinus membrane thickness and lateral wall thickness. Furthermore, there was a strong association between lateral wall thickness and female patients, and premolar areas had thicker sinus membranes than molar areas. As a result, the current study’s findings will aid clinicians during clinical procedures. We believe the data from the present study is necessary for the further development of a clinically applicable set of formulae to determine the success for various surgical procedures in the maxillofacial region involving maxillary sinus. Additionally, this current research design will pave the way for future studies.

## Supporting information

S1 Dataset (XLSX)
